# Current Fertility Preservation Steps in Young Women Suffering from Cancer and Future Perspectives

**DOI:** 10.3390/ijms25084360

**Published:** 2024-04-15

**Authors:** Alicia Marco, Marta Gargallo, Jesús Ciriza, Ariella Shikanov, Laura Baquedano, Javier García Pérez-Llantada, Clara Malo

**Affiliations:** 1 Faculty of Medicine, University of Zaragoza, 50018 Zaragoza, Spain; aliciamarcogabarre@gmail.com; 2 Institute for Health Research Aragón (IIS Aragón), 50009 Zaragoza, Spain; martagargallo13@gmail.com (M.G.); jeciriza@unizar.es (J.C.); 3 Tissue Microenvironment (TME) Lab, Aragón Institute of Engineering Research (I3A), University of Zaragoza, 50018 Zaragoza, Spain; 4 Department of Biomedical Engineering, University of Michigan, Ann Arbor, MI 48109, USA; shikanov@umich.edu; 5 Department of Obstetrics and Gynecology, University of Michigan, Ann Arbor, MI 48109, USA; 6 Cellular and Molecular Biology Program, University of Michigan, Ann Arbor, MI 48109, USA; 7 Department of Gynecology, University Hospital Miguel Servat, 50009 Zaragoza, Spain; lbaquedanome@hotmail.com; 8 Hospital Viamed Montecanal, 50012 Zaragoza, Spain; consulta@llantada.com

**Keywords:** fertility preservation, ovarian tissue, cancer treatment, cryopreservation, follicular loss

## Abstract

Childhood cancer incidence, especially in high-income countries, has led to a focus on preserving fertility in this vulnerable population. The common treatments, such as radiation and certain chemotherapeutic agents, though effective, pose a risk to fertility. For adult women, established techniques like embryo and egg freezing are standard, requiring ovarian stimulation. However, for prepubescent girls, ovarian tissue freezing has become the primary option, eliminating the need for hormonal preparation. This review describes the beginning, evolution, and current situation of the fertility preservation options for this young population. A total of 75 studies were included, covering the steps in the current fertility preservation protocols: (i) ovarian tissue extraction, (ii) the freezing method, and (iii) thawing and transplantation. Cryopreservation and the subsequent transplantation of ovarian tissue have resulted in successful fertility restoration, with over 200 recorded live births, including cases involving ovarian tissue cryopreserved from prepubescent girls. Despite promising results, challenges persist, such as follicular loss during transplantation, which is attributed to ischemic and oxidative damage. Optimizing ovarian tissue-freezing processes and exploring alternatives to transplantation, like in vitro systems for follicles to establish maturation, are essential to mitigating associated risks. Further research is required in fertility preservation techniques to enhance clinical outcomes in the future. Ovarian tissue cryopreservation appears to be a method with specific benefits, indications, and risks, which can be an important tool in terms of preserving fertility in younger women.

## 1. Introduction

Fertility preservation is defined as the implementation of medical strategies and laboratory procedures aimed at safeguarding the genetic offspring of adults or children at risk of infertility. It is a medical measure directed toward preventing and safeguarding future fertility [1]. In young women with cancer, it has gained importance in oncological care.

Approximately 120,000 girls aged from 0 to 19 years old are diagnosed with cancer each year. The burden of childhood cancer is strongly related to the wealth of a country, with a high incidence in high-income countries but higher mortality in low-income countries [2]. The most common tumors in this age group are hematological, mainly leukemias, followed by tumors of the central nervous system, especially neuroblastoma. Bone tumors, such as Ewing’s sarcoma, also have a significant impact. Although these tumors do not directly affect ovarian tissue, some treatments, such as pelvic radiation and certain gonadotoxic chemotherapeutic agents like Cyclophosphamide or Busulfan [3,4], despite being effective, can have adverse consequences on fertility. Some medications and radiation therapies can affect ovarian tissue, damage oocytes, and reduce reserves [5,6]; they have been shown to be highly gonadotoxic, raising concerns about fertility preservation in these patients [7]. Research has focused on finding the best way to preserve the fertility of these girls, considering their age, characteristics, and needs, as in many cases, the impact is not limited only to future reproductive capacity but also affects physiological pubertal development.

**Figure 1:**
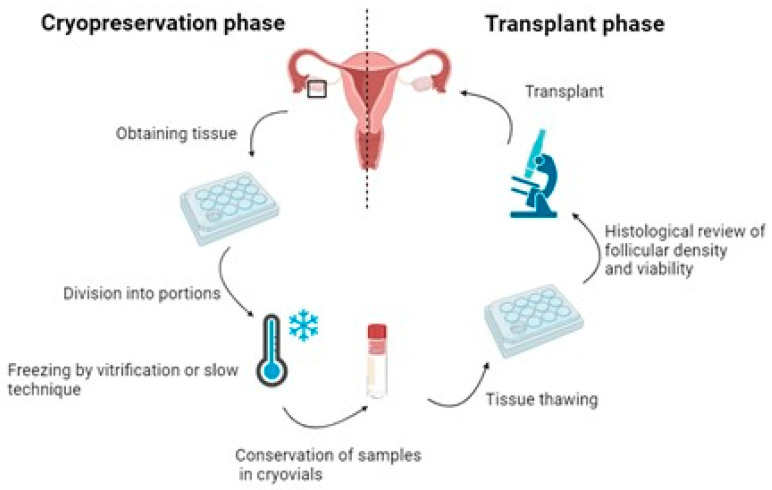
Diagram of the ovarian tissue freezing process. Created by BioRender. Figure reproduced from Chen et al. 2022 [9].

Currently, the main techniques for preserving fertility in adult women include embryo and egg freezing. These methods require ovarian stimulation treatment, involving prior hormonal maturation and, thus, the development of the hypothalamic–pituitary–ovarian axis. However, for prepubescent girls, whose lack of hormonal maturation and the need to initiate oncological treatment immediately pose challenges, the only viable option is ovarian tissue freezing with the potential for future transplantation. Unlike conventional techniques, this alternative does not require prior hormonal preparation and can be performed at any time [8] (Figure 1).

The cryopreservation and subsequent transplantation of ovarian tissue have yielded promising results in the field of fertility preservation. The most frequent malignancies considered as indications are leukemias, myeloproliferative or myelodysplastic diseases, neurological neoplasms and sarcomas, and other non-malignant diseases such as Turner syndrome or galactosemia. Indications include treatment with alkylating agents, pre-allograft and autologous hematopoietic stem cell conditioning, ovarian-focused radiotherapy, and gonadectomy. Ovarian tissue cryopreservation is discouraged in acute leukemia cases. Since the implementation of this technique, approximately 200 live births have been recorded, with a notable percentage (15–20%) of cases performed with ovarian tissue cryopreserved from prepubescent girls [10]. According to recent data, 1019 ovarian tissue cryopreservation procedures have been performed in children and young adults with cancer (0–20.4 years), of whom 298 were younger than 13 years. Eighteen of them received an ovarian tissue (OT) transplant in adulthood, resulting in eleven pregnancies, of which nine resulted in a live birth [11,12]. Despite these encouraging results, two fundamental limitations persist in the methodology. First, in adult women, a loss of over 50% of follicles has been observed due to ischemic and oxidative damage during tissue transplantation. On the other hand, there is concern related to the possibility of reintroducing cancer cells or oncologic tissue present in the frozen sample [13]. 

Thus, it is imperative to advance in optimizing the ovarian tissue-freezing process and explore alternatives to transplantation, such as the development of in vitro systems for the growth and maturation of primordial follicles to yield fertilizable oocytes. These strategies could eliminate the need for tissue transplantation, thereby mitigating associated risks.

Throughout the 18th and 19th centuries, the American surgeon Robert Morris stood out as a pioneer in the field of ovarian tissue transplantation, demonstrating that uterine atrophy was reversible and that menopausal symptoms could be alleviated through these transplants [14]. In 1994, the first autotransplantation studies after cryopreservation were conducted in sheep, successfully restoring ovarian function and enabling natural conception through the reintroduction of previously extracted and frozen tissue fragments. These investigations extended to different animal species, demonstrating the feasibility of recovering both endocrine and ovarian function long-term after the cryopreservation and transplant process [8,15].

In 1999, the first human ovarian tissue autotransplantation took place, marking a significant success that spurred the initiation of research aimed at improving the technique [11]. In 2004, the first live birth was recorded from a woman who turned to this fertility preservation technique due to Hodgkin’s lymphoma [16].

However, it was not until 2013 that the first case was documented in which puberty was induced in a girl who experienced primary ovarian failure after receiving chemotherapy and radiotherapy treatment. Diagnosed with Ewing’s sarcoma at the age of 9, a portion of her left ovary was cryopreserved. After complete disease remission four years later, the patient experienced severe primary ovarian failure with postmenopausal levels of follicle-stimulating hormone (FSH). By transplanting two tissue samples into the remaining ovary, which had suffered the consequences of treatment and had precarious vascularization, Tanner stage 4 was achieved, maintaining regular menstruation for the next 4 months. Effective ovarian function was achieved for 19 months with just two fragments [17].

These advancements continue to drive the ongoing quest to optimize the cryopreservation technique and enhance its success rates, opening new possibilities in both the medical and scientific research realms.

## 2. Anatomy and Physiology of Ovarian Tissue

**Figure 2:**
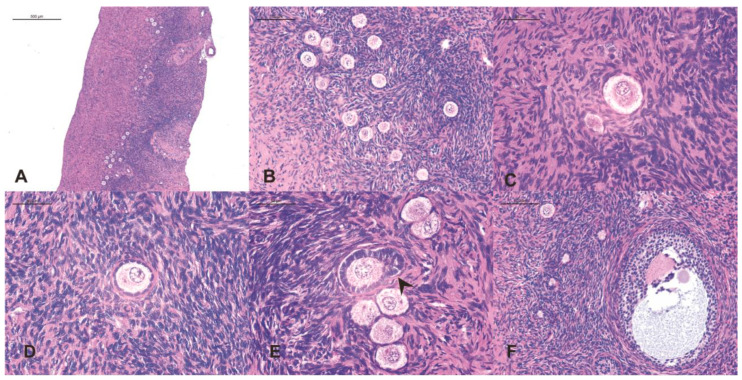
Hematoxylin eosin (HE) staining of fresh human ovarian cortex tissue and follicles. ((**A**) ×50 and (**B**) ×100). Follicle stages were classified using the following criteria: primordial (one layer of flattened granulosa cells), ×200 (**C**); primary (one layer of fully expanded, cuboidal granulosa cells), ×200 (**D**); secondary indicated with an arrow (two distinct layers of expanded, granulosa cells), ×200 (**E**); antral (with visible > antrum), ×100 (**F**) [19].

Histologically, the ovary is divided into the outer cortex and the smaller medulla. The medulla consists of loose connective tissue, primarily housing the vascular component of the organ. On the other hand, the cortex contains the stroma along with ovarian follicles [18]. The latter are structures composed of a female germ cell surrounded by follicular cells, and they prevail in their state of primordial follicles, representing approximately 90% of the cortical follicular population. Other types of follicles (primary, secondary, and mature) emerge during puberty with the onset of menstrual cycles, resulting from the maturation of primordial follicles. The significance of the primordial follicle lies in its small size, low water content, and low metabolic requirement, attributes that confer on them a greater resistance to stress and, therefore, a higher capacity for survival. Additionally, primordial follicles constitute the main cellular population of the prepubertal ovary, further enhancing the utility of cryopreservation in girls, who possess a considerably larger reserve [18] (Figure 2).

In a study conducted by Christianson et al. [20], the implications of a higher reserve of primordial follicles were explored by evaluating follicular density in six patients under 18 years old (prepubertal and pubertal) slated for ovarian tissue transplantation. These patients, mostly affected by Hodgkin’s lymphoma, underwent ovarian tissue biopsy concurrently with the placement of a catheter for chemotherapy. The tissues were frozen for several days and then transplanted into mice, with samples extracted after a specified period to assess tissue status. A higher follicular density was observed in prepubertal women compared to pubertal ones, demonstrating a relationship between age, density, and follicular maturation state. This finding supports the notion that the duration of post-transplant ovarian function is directly linked to follicular type and density. The research suggests that having prior information about the patient’s ovarian reserve can estimate transplant success and duration. In cases of limited primordial follicle reserve, a more restricted graft functionality is anticipated. However, it is crucial to interpret these results with caution due to the small sample size of patients and the brief tissue-freezing period compared to usual standards in this technique [20,21].

The hormonal function of ovarian tissue is crucial for reproductive and metabolic health, and its preservation is essential in girls who are subjected to treatments that can affect it. Ovarian tissue cryopreservation offers a significant advantage over other fertility preservation techniques by providing the possibility of restoring hormonal function. This aspect becomes particularly relevant since often both girls and their families are more concerned about age-appropriate sexual maturation than considerations related to future reproduction. It is essential to note that ovarian tissue plays a fundamental role not only in gamete production but also in the synthesis of sex hormones, which, in addition to initiating pubertal development, fulfill crucial metabolic functions such as maintaining cardiovascular and bone health. This procedure seems to have some advantages and appears to be a simple and low-risk treatment to induce puberty. However, there are also some disadvantages to consider. Ovarian tissue transplantation cannot prevent and replace hormonal replacement therapy as the tissue survival is limited, there is a rapid hormone production due to an imbalanced hypothalamic–pituitary–ovary axis, and this sudden and overt increase in estrogen production seen a few months after tissue transplantation could induce accelerated pubertal development, leading to side effects. This endocrine restoration is paramount, as it helps avoid the risk of girls experiencing a postmenopausal condition at premature ages [22]. In this context, anti-Müllerian hormone (AMH) has been identified as a marker of ovarian function, playing a significant role in the regulation of follicular development and selection for ovulation [23].

Although attempts are sometimes made to preserve one of the ovaries to protect this function, if the treatment adversely affects the organ, ovarian tissue transplantation emerges as the only viable option to address this issue. In a longitudinal study spanning 10 years conducted in 5 girls, it was observed that all patients achieved the restoration of their endocrine function approximately by week 11 post-transplant. The graft durations reached up to 7 years, although in some cases, second transplants were required to achieve such restoration [24].

## 3. Steps in the Current Fertility Preservation Protocols

Fertility preservation through tissue extraction and freezing is a crucial aspect of reproductive medicine, particularly for cancer patients [25]. Next, the fertility preservation steps are described: ovarian tissue extractions, freezing methods, and transplantation. 

### 3.1. Ovarian Tissue Extraction

Until recently, common practice involved total ovarian extraction, fragmenting it into thin slices for subsequent storage. However, it was observed that in many cases, the risk of primary ovarian failure was limited, raising questions about the justification of the risk/benefit associated with complete gonad removal. Consequently, current strategies are adapted to extract a piece of tissue, considering each patient’s probability of ovarian failure, reserving total ovarian removal for situations where the woman presents with a risk exceeding 80% [26].

Risk calculation is based on factors such as age, cancer type, planned treatment, and its duration. In this context, Clark et al. examined the possibility of early ovarian failure in childhood cancer survivors, defining such failure as the loss of ovarian function within 5 years of diagnosis or the absence of menarche at 18 years old. Evaluated parameters for risk calculation included radiation, dosage, location, age, menarche, cancer type, exposure to chemotherapeutic agents (with special attention to alkylating agents), and whether hematopoietic stem cell transplantation had been performed or not. It was concluded that the best way to calculate the risk of ovarian failure was to determine ovarian damage based on the dose and amount of chemotherapy and radiation received [22]. As an example, it was highlighted that pelvic area radiation at doses of 20–30 Grays could induce primary ovarian failure [27]. However, in prepubescent patients, a lower risk of primary ovarian failure was observed compared to adults, suggesting the application of more conservative approaches [28].

**Figure 3:**
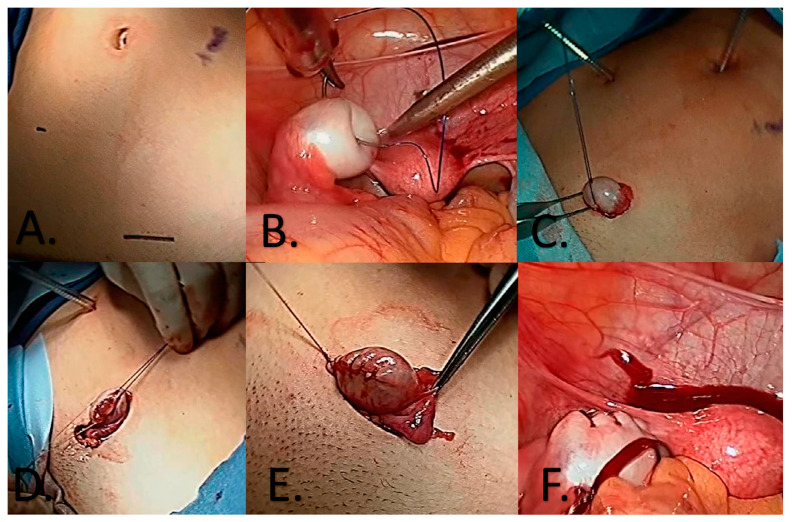
Extracorporeal ovarian extraction procedure [26]. (**A**) Port sites locations; 5 mm at the umbilicus, 5 mm in the right lower quadrant, 12 mm in the left suprapubic area. (**B**) Placement of the traction suture through the left ovary. (**C**) Removal of the 12 mm trocar from the field and utilizing the traction suture to deliver the left ovary to an ex vivo position. May require extension of the incision. (**D**) With suture stays and intermittent vascular control, a wedge resection of the left ovary performed with scalpel. (**E**) Following removal of the ovarian tissue, hemostasis is obtained with electrocautery and the ovarian capsule is closed with running vicryl suture. (**F**) Ovary is placed back in its native position and hemostasis ensured.

For ovarian tissue retrieval, the least invasive option involves laparoscopy, in which the ovary is manipulated using monofilament traction sutures to position it extracorporeally. This allows for the extraction of necessary tissue without the need for complete ovary removal, facilitating the subsequent reintegration of the remaining ovarian tissue into its functional position with ease [26]. This procedure seeks to preserve ovarian tissue promptly, without delaying the initiation of chemotherapy treatment [29] (Figure 3). The viability of extracting and preserving the entire ovary was also studied, but cryopreservation results were significantly inferior, increasing the risk of transplanting malignant cells [30].

### 3.2. Freezing Methods

The storage of cells and tissues at low temperatures for preservation purposes has been a subject of research since the 19th century. Cryopreservation is a crucial method for long-term cellular preservation, and recent advances minimize cellular damage. However, this method has significant limitations, as cells in our bodies are not inherently designed to withstand extremely low temperatures. Since approximately 80% of tissue mass is composed of water, freezing can trigger biochemical and structural consequences that are harmful to cells [31].

Two main effects result from this process. First, the formation of ice crystals can disrupt cellular membrane components, altering their structure. Second, there is an increase in solute concentration in the remaining liquid phase, leading to highly toxic osmotic stress [32]. To avoid these adverse effects, it is essential to use cryoprotectants and have specific knowledge about each type of cell and its particular characteristics. Differences in cellular size and composition will determine how they respond to temperature reduction, emphasizing the need for personalized approaches for the successful preservation of various cell types.

Cryoprotectants, also known as cryoprotective agents, are water-soluble substances with low toxicity that play a crucial role in reducing osmotic stress experienced by cells during the freezing process. These compounds can be classified based on their nature or their penetration capacity. In terms of nature, various categories are distinguished, including alcohols such as ethanol, ethylene glycol (EG), propanediol (PROH), and glycerol; sugars such as glucose, lactose, sucrose, and saccharose; and organic sulfurs represented by dimethyl sulfoxide (DMSO). Based on penetration capacity, cryoprotectants can be divided into two categories: permeating agents such as glycerol, DMSO, EG, and PROH, which have a low molecular weight and can pass through the lipid bilayer of the cell membrane; and nonpermeating agents such as sugars (sucrose, trehalose, and raffinose) and macromolecules (Ficoll and polyvinylpyrrolidone), as well as proteins and lipoproteins, which remain in the extracellular solution, for they are large molecules and help to promote controlled cell dehydration. Therefore, it is essential not only to understand the types of cryoprotectants and their properties but also to explore other substances that can optimize the process [33].

In addition to cryoprotectants, other substances have been studied that can be added to the freezing medium to improve results. Albumin, in addition to its oncotic regulation capacity, can contribute to pH control in the medium. Numerous studies have highlighted the significance of antioxidants such as resveratrol [34], melatonin [35], Vitamin E [36], and catalase [37] in mitigating cryopreservation transplantation-induced injuries in ovarian tissue.

**Figure 4:**
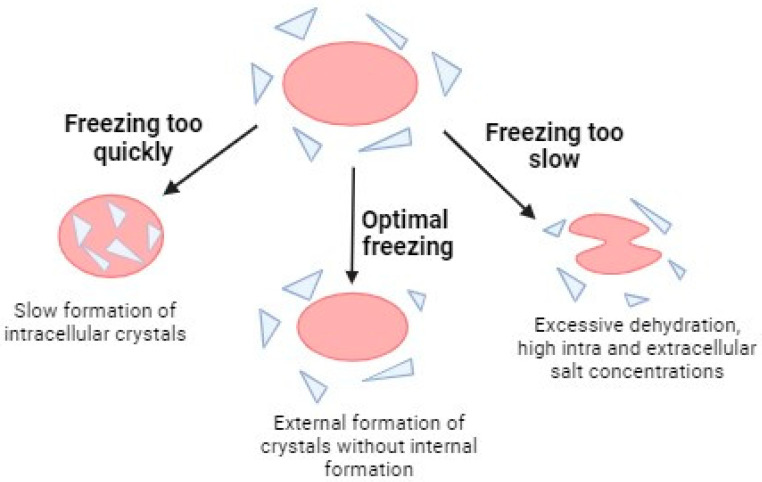
Scheme of issues associated with different freezing rates. Created in BioRender. Figure reproduced from Murray and Gibson (2022) [40].

In cryopreservation procedures, two approaches are primarily employed: slow freezing and rapid freezing or vitrification. In slow freezing, tissues are exposed to low doses of cryoprotectants and stored in a programmable freezer or freezing boxes (cell freezer), where the temperature decreases gradually [38]. The goal of slow freezing is to allow water to exit gradually from the cells, minimizing the formation of intracellular crystals. However, this process has its limits, as an excessively slow freezing rate can result in excessive cellular dehydration and, therefore, irreparable cell damage. It is crucial to determine the optimal freezing rate, specific to each cell type, considering that the effective freezing curve for some cells may be harmful to others and vice versa [39] (Figure 4).

In the case of ovarian tissue, which involves various cell types, it has been observed that to effectively carry out slow freezing, it is recommended to program the freezer with different freezing ramps that gradually reduce the temperature. An example of this procedure includes a first stage with a temperature decrease from 16 °C to −7 °C at a rate of 2 °C/min, a second stage maintaining the temperature at −7 °C for 10 min, a third stage in which cooling from −7 °C to −30 °C occurs at a rate of 0.3 °/min, a fourth stage of cooling from −30 °C to −150 °C at a rate of 50 °C/min, and a fifth stage where the temperature is maintained at −150 °C for approximately 30 min before transferring the tissue to liquid nitrogen storage [41].

A popular protocol for slow freezing uses a medium composed of 5% serum albumin, 10% DMSO, and medium 199. After immersion in 5% DMSO for 15 min and then in 10% DMSO for 15 min, the samples are placed in a freezer programmed according to the following protocol: a first cooling stage from 10 to −8 °C at 1 °C/min, a second cooling stage to −15 °C at 2.5 °C/min, a third stage of maintenance for 9 min at −15 °C, a fourth cooling stage to −35 °C at 0.5 °C/min, a fifth cooling stage to −60 °C at 5 °C/min, a sixth cooling stage to −120 °C at 10 °C/min, and, finally, the samples are transferred to liquid nitrogen at −196 °C [42,43].

On the other hand, vitrification involves exposing tissue samples to cryoprotectants in high concentrations, followed by rapid freezing at low temperatures. The transition from room temperature to −196 °C occurs extremely rapidly, avoiding the need for osmotic balance between intra- and extracellular spaces. In this process, the solution solidifies quickly due to cooling, acquiring a glassy state due to increased viscosity. This rapid temperature transition avoids “chilling injury”, the critical window of highest susceptibility to cold damage, which occurs between 15 °C and −5 °C. However, a key challenge associated with vitrification lies in the potential toxicity of high doses of cryoprotectants, which can be counterproductive. To address this issue, a combination of cryoprotectants with different characteristics is used to reduce toxicity. The concurrent use of cryoprotectants with anti-frozen proteins (AFPs) has also been explored, which could decrease the cryoprotectant dose without compromising safety in terms of freeze injuries [44].

Commercial vitrification kits designed for freezing oocytes, embryos, and ovarian tissue exist, although the latter is less common. Commercial kits typically consist of several solutions with staggered concentrations of cryoprotectants to allow for the sequential freezing of samples. An example is the Kitazato brand kit [45], which incorporates components such as hydroxypropylcellulose (HPC), trehalose, DMSO, and gentamicin.

**Figure 5:**
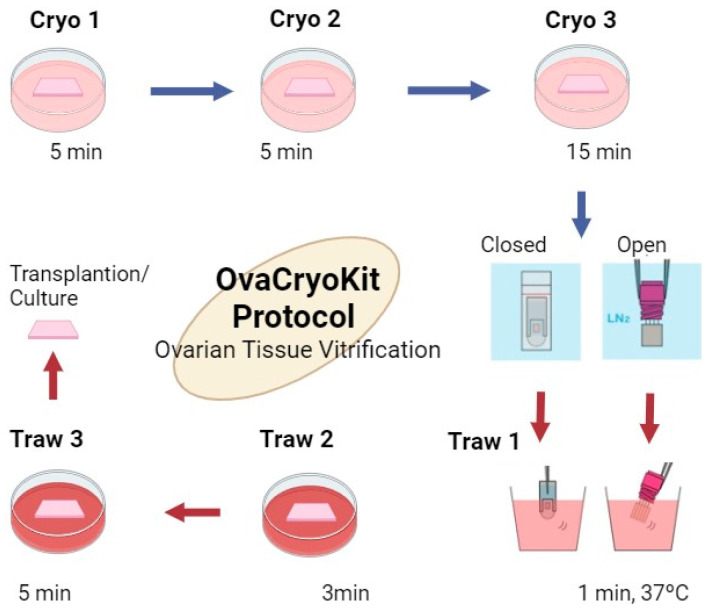
Operation scheme of the Kitazato kit for ovarian tissue cryopreservation. Created in BioRender. Figure reproduced from Kitazato Coorporation [45].

The ovarian tissue cryopreservation method proposed by the Kitazato kit indicates variations depending on whether it is performed in a closed or open system (Figure 5). Although this kit is not yet commercially available in the European Union, it represents a notable example of advances in vitrification techniques.

In recent years, vitrification has emerged as a prominent method, especially in the freezing of oocytes and embryos, by avoiding the problematic formation of ice crystals without compromising viability and reserve outcomes. This approach has been routinely adopted in fertility clinics. However, the complexity and size of ovarian tissue, comprising various cell types, poses challenges in implementing vitrification protocols, and further research is still required to determine the optimal way to carry it out.

In this context, two reviews comparing the differences between slow freezing and vitrification in ovarian tissue have been published, analyzing recent studies. A meta-analysis conducted in 2017 by Shi et al. (2017) revealed not only no statistically significant differences in follicular density and the percentage of primordial follicles between both techniques but also that vitrification showed a better preservation of stromal cells and lower DNA damage [46]. On the other hand, in the review by Kometas et al. (2021), it was observed that there were no morphological differences, but a significant limiting factor was highlighted: many women who undergo ovarian tissue freezing ultimately do not proceed to thawing and transplantation, preventing conclusive results on the technique [47]. A study conducted at the Hospital de la Fe in Valencia, Spain, revealed that transplanting human ovarian tissue into immunocompromised mice resulted in fewer primordial follicles and lower follicular reserve with slow freezing compared to vitrification [42].

Although slow freezing is the most standardized method in Europe for ovarian tissue, the results obtained in these studies, combined with the fact that vitrification is a more efficient process in terms of resources and time, suggest the possibility of considering vitrification as an alternative technique for ovarian tissue preservation in the future [8]. Slow freezing is by far the most preferred technique worldwide with more than 200 estimated births after slow freezing and transplantation, whereas only 4 births following the vitrification technique are described [48]. More extensive research in this direction is needed to determine the safest and most effective way to perform this procedure.

### 3.3. Ovarian Tissue Thawing and Transplantation 

The thawing and subsequent transplantation process of ovarian tissue constitute a critical point at which tissue viability may be compromised. The thawing methodology varies depending on the type of freezing and cryoprotectants used. In the case of vitrification, one method involves leaving the tissue for 2 min at room temperature in a thawing solution, followed by immersion in a water bath at 37 °C [30,32,49]. For slow freezing, a common thawing protocol involves placing the vial of frozen tissue samples in water at 90–95 °C, until the ice is melted. Then, the samples are subjected to various rehydration solutions at 25 °C [41]. In both methods, it is crucial to gradually remove the cryoprotectant after thawing, as abrupt withdrawal could cause cell expansion followed by shrinkage, resulting in significant damage [31]. Other methods, such as the use of infrared laser pulses, are being explored to simplify the thawing process of ovarian tissue, oocytes, and embryos [29]. Despite these investigations, temperature changes during thawing inevitably lead to a loss in the viability of tissues.

The transplant location can be orthotopic or heterotopic, depending on whether it is performed in the physiological ovarian space or in other areas such as the peritoneum, abdominal wall, or even the forearm, a technique that is currently being investigated for minimally invasive transplant approaches [50]. The tissue location has not demonstrated an impact on the onset of hormonal function, with total endocrine functions being restored between weeks 10–20 after transplantation [24]. However, further research is needed to understand the effectiveness of fertility restoration in heterotopic transplants, as fertility rates in these cases have been observed to be less promising than in orthotopic transplants [13]. Additionally, it has been observed that the closer the orthotopic transplant is to the fallopian tubes, the higher the probability of achieving natural conception, while more distant transplants are more likely to require assisted reproductive techniques [30].

One of the most significant challenges in ovarian tissue transplantation is the notable follicular loss experienced. It is estimated that this loss reaches around 50% in adult women, although in preadolescents, a decrease is observed due to the prevalence of primordial follicles, which show greater resistance to cryogenic injuries [51]. The decrease in ovarian reserve originates from two main mechanisms: direct cell death due to freezing damage, ischemia, or oxidation; and the massive activation of follicles, known as the follicular burnout phenomenon or “follicular exhaustion”.

**Figure 6:**
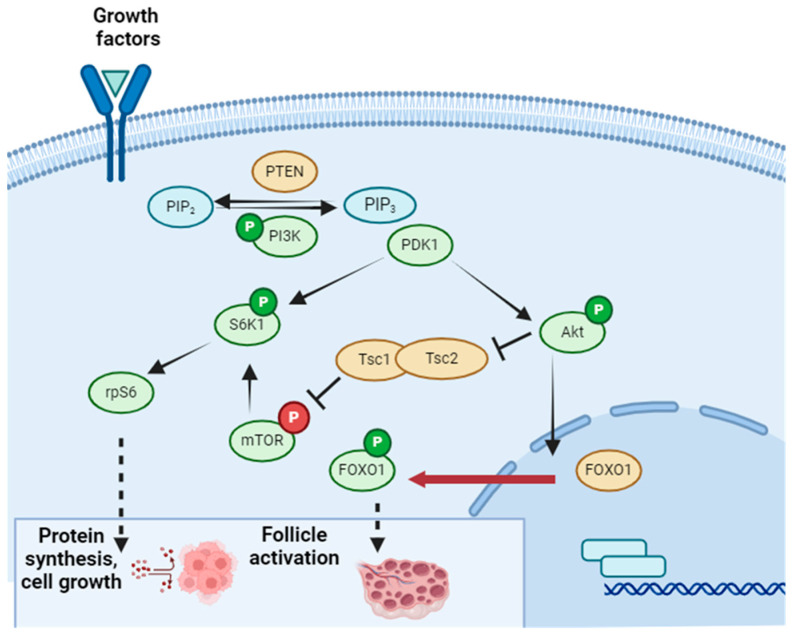
PI3K Pathway. Created in BioRender. Figure reproduced from Dolmans et al. 2021 [51].

One of the pathways involved in the burnout phenomenon is the PI3K pathway [51]. This pathway is essential in nutrient metabolism, cell survival, growth, and apoptosis. The activation of its effector, Akt, is regulated by the inhibition of the enzyme PTEN and the activation of another enzyme called PI3K. When activation prevails, Akt phosphorylates, triggering phosphorylation cascades, such as FOXO1 and mTOR. FOXO1 acts as a transcription factor in primordial follicles, inhibiting their growth. Its phosphorylation translocates FOXO1 to the cytoplasm, losing its inhibitory function and activating follicular growth. Although mTOR is essential in cell growth regulation, its involvement is believed to occur later, once follicles have reached a certain level of maturity, and it would not be implicated in early massive activation [52] (Figure 6).

Under normal conditions, granulosa cells express PTEN, maintaining follicular quiescence. However, in situations of oxidative stress, PI3K is overexpressed, deactivating that inhibition. Numerous studies have corroborated that this pathway plays a fundamental role in massive follicular consumption after transplantation as a cellular defense response [50,51,52]. Faced with hypoxia, the tissue activates its cells to survive.

The role of other pathways, such as the Hippo pathway, has also been explored. This pathway is essential for regulating tissue size, acting through inhibitory regulatory factors that, through a cascade of kinases, phosphorylate the pathway to keep its effectors YAP/TAZ inactive. If the pathway is damaged, levels of these effectors increase, promoting cell growth and proliferation. Studies evaluating the involvement of this pathway in the burnout process have yielded diverse results. For example, a study by Masciangelo et al. observed an increase in YAP/TAZ levels after the transplantation of human ovarian tissue into immunocompromised mice, confirming the contribution of this pathway. However, subsequent research has suggested that its role may not be as significant [50].

Other pathways such as cytokine–cytokine receptor interaction and the MAPK signaling pathway were associated with mammalian reproduction. Most of the differentially expressed genes participating in the cytokine–cytokine receptor interaction and the MAPK signaling pathway were upregulated, which indicated that these two pathways were activated in human ovarian tissues after vitrification/warming. Other pathways associated with reproduction are the ECM–receptor interaction, focal adhesion, and Jak–STAT. Gu et al. [53] demonstrated that the cryopreservation process can activate or deactivate these physiological functions. However, once ovarian grafts were able to support complete oocyte growth, the dysregulation of genes could be associated with a compensatory mechanism to maintain ovarian viability after vitrification, which qualitatively did not impair oogenesis.

In addition, past studies have discovered higher levels of DNA fragmentation, gene underexpression, metabolic and secretory issues, significant telomere shortening, and altered senescence markers and mitochondrial structures. Zhou et al. [54] performed an interesting study about the effect of the two-time cryopreservation of ovarian tissue and its effect on RNA transcriptomics. Parathyroid hormone synthesis, secretion, and action, MAPK signaling pathways, cellular senescence, cell cycles, TNF signaling pathways, basal cell carcinoma, FoxO signaling pathways, and p53 signaling pathways were affected after double cryopreservation. p53 is a senescence marker that was downregulated after cryopreservation in human ovarian tissues [55].

In any case, reducing the ischemia period, combined with antioxidants or growth factors such as VEGF to optimize tissue vascularization, could minimize both direct cell damage and the activation of these pathways. Substances such as fibroblast growth factor have been studied, and their transplantation with ovarian tissue in immunocompromised mice demonstrated a higher density of primordial follicles [56]. Another promising alternative is stem cells, specifically adipose tissue precursor cells, which significantly favor angiogenesis. In studies with immunocompromised mice, lower hypoxia was observed, resulting in a higher ovarian reserve and a more physiological follicular distribution [57].

Recently, the protein S1P (sphingosine-1-phosphate) has been investigated in animal studies. This pathway, inhibiting cell death, already used against apoptotic death induced by radiation and chemotherapy, has the potential to accelerate neovascularization, reduce hypoxia, and preserve follicular density after transplantation [11]. Additionally, an increase in stromal cell numbers has been observed, providing benefits to the graft [58]. Other approaches have explored the possibility of using engineered endothelial cells, which constitutively express anti-Mullerian hormone (AMH). This strategy has been associated with accelerated post-transplant vascular reperfusion and the maintenance of quiescent follicle reserves. This approach is based on the observation that growing follicles, like secondary ones, naturally express this hormone to inhibit the growth of primordial follicles, thus preserving the ovarian reserve. An environment rich in AMH decreases follicle sensitivity to follicle-stimulating hormone (FSH), contributing to halting follicular growth [59].

Additionally, the use of minimally invasive surgical techniques could effectively address the ischemic problem by minimizing vascular damage at the transplant site. The application of surgical robots offers greater precision compared to conventional laparoscopic techniques, potentially reducing the time between thawing and transplantation [60]. This innovative approach could not only improve procedure efficiency but also mitigate ischemic effects associated with ovarian tissue transplantation, thus promoting better clinical outcomes.

## 4. Risks Associated with Ovarian Tissue Transplant

One of the primary concerns for researchers is the potential reintroduction of cancer cells from the primary tumor to patients in complete remission through frozen tissue. This concern is particularly pronounced in cancers directly affecting gonadal tissue, such as hematological tumors. Studies conducted by Dolmans et al. [61] and Meirow et al. [62] have found evidence of contamination with leukemic cells in cryopreserved ovarian tissue, with the latter emphasizing the need for sensitive markers to detect minimal residual disease. On the other hand, the study by Soares et al. [63] demonstrated the possibility of eliminating malignant cells from cryopreserved ovarian tissue in leukemia patients, offering hope for fertility restoration. These studies underscore the importance of thoroughly analyzing the samples in the laboratory before freezing, using various markers to ensure the absence of contamination in the sample (preoperative imaging, histology and immunohistochemistry, and PCR). Preoperative imaging, histology and immunohistochemistry are unable to detect smaller lesions that may contain malignant cells. RT-PCR is a highly sensitive method but requires a specific sequence.

A notable example is the case of a girl who experienced primary ovarian failure as consequence of hematopoietic progenitor transplantation to treat her leukemia. The tissue was collected just after the first conditioning treatment and subsequently frozen using the slow technique. Different tissue stains were performed to ensure the absence of cancer cells. Ten years after preservation, tissue transplantation was carried out, and the patient conceived and gave birth to two healthy children, showing no signs of leukemia relapse. This case illustrates that, with proper analyses, the likelihood of transferring cancer cells through frozen tissue can be practically negligible [64].

However, there are situations where the tissue may be contaminated and unsuitable for transplantation. Various intervention strategies are being explored for these cases, from simpler approaches, such as applying low-dose chemotherapy before transplantation to reduce the risk of transferring cancer cells, to more advanced and ambitious methods, such as culturing primordial follicles in the laboratory until reaching the mature egg stage, known as in vitro follicular growth. The latter method presents additional advantages, such as avoiding follicle loss due to burnout, ischemia, or recruitment, more efficiently utilizing the primordial follicular population, and eliminating the need for a second intervention for transplantation, among other benefits. However, in vitro follicular growth faces significant challenges and has low success rates due to the complexity of the folliculogenesis process, making it still considered experimental.

## 5. In Vitro Oogenesis from Stem Cells and In Vitro Follicle Growth Protocol for Avoiding Transplant

Over the past decade, great progress has been made in the establishment of in vitro culture models of oogenesis. Recently, new reports have introduced the possibility of obtaining functional gametes derived in vitro from stem cells in mice [65,66,67]. In humans, the specific molecular niche that leads to correct oogenesis is less understood. There are great attempts to induce meiosis and haploid cells using embryonic stem cells (ESCs) and induced pluripotent stem cells (iPSCs) under different induction conditions [68,69]. However, the induction is inefficient.

In vitro follicle growth, as an alternative to transplantation, has shown promising advances in fertility preservation, as highlighted by Yang et al. [70] and Telfer et al. [71]. Emphasis is placed on optimizing cultivation conditions in conducted research, as metabolic dynamics and necessary nutrients vary across different stages of follicular development. This indicates that during the process of follicle growth and development, there are changes in how cells metabolize nutrients and in the specific nutrients required for each stage. This knowledge is pertinent in the context of in vitro follicle culture, where understanding and adapting to these variations are crucial for the success of the process and, consequently, for successful fertility preservation [70].

Additionally, the development of in vitro systems to support the complete growth of follicles from the earliest primordial stages to maturity has been analyzed as an alternative. This advancement holds various therapeutic applications, including the production of oocytes for assisted reproductive technologies, the assessment of toxicological effects on germ cell development, the examination of cryopreserved ovarian tissue before transplantation, and the provision of an experimental model to address basic scientific questions about human oocyte development [71]. Other authors further endorse this potential. Xiao et al. [72] demonstrated the production of meiotically competent metaphase II oocytes after in vitro maturation, while Marin et al. [73] explored the potential of in vitro growth for a wide range of patients, delving into the detailed evaluation of fundamental criteria, such as cellular morphology, cellular viability, hormonal production, and gene expression, to measure the efficiency and quality of follicle growth during in vitro gametogenesis (IVG) [73].

The successful development of human oocytes from the early follicular stages to maturation and in vitro fertilization has revolutionized fertility preservation practices. This achievement, demonstrated in mice where in vitro-cultivated oocytes from primordial follicles resulted in live offspring, signifies a remarkable breakthrough. Several groups are actively developing systems to support the growth and development of oocytes from the human ovarian cortex [74]. Despite these advancements, challenges persist, including the complexity of the in vivo microenvironment, precise hormonal regulation, issues of incomplete maturation, and ethical and legal considerations [57]. However, the ongoing development of in vitro follicle growth has the potential to revolutionize fertility preservation, marking significant progress in scientific and medical research.

## 6. Summary

Fertility preservation techniques, such as ovarian tissue freezing, have become crucial in the oncological care of young women with cancer, given the rising cases of childhood cancer and the imperative to safeguard future fertility. Ovarian tissue extraction through laparoscopy emerges as a less invasive and secure option for oncology patients seeking to preserve fertility before undergoing treatment. The cryopreservation process, whether through slow freezing or vitrification, presents challenges regarding the toxicity of cryoprotectants, demanding further research to optimize these techniques. The choice of ovarian tissue transplant location, whether orthotopic or heterotopic, and the need to enhance tissue viability post-thawing are key areas requiring additional study to advance successful fertility preservation in oncology patients. The prospect of cultivating follicles in vitro as an alternative to transplantation holds promise in fertility preservation. However, it still encounters significant challenges, including the intricacies of the process and ethical and legal considerations.
